# Saponin Extracts Utilization as Dietary Additive in Ruminant Nutrition: A Meta-Analysis of In Vivo Studies

**DOI:** 10.3390/ani14081231

**Published:** 2024-04-19

**Authors:** Yulianri Rizki Yanza, Agung Irawan, Anuraga Jayanegara, Fitri Ramadhani, Adib Norma Respati, Ainissya Fitri, Cecep Hidayat, Vincent Niderkorn, Adam Cieslak, Malgorzata Szumacher-Strabel, Rahmat Hidayat, Ujang Hidayat Tanuwiria

**Affiliations:** 1 Department of Animal Nutrition and Feed Technology, Faculty of Animal Husbandry, Universitas Padjadjaran, Jatinangor, Sumedang 45363, West Java, Indonesia; r.hidayat@unpad.ac.id (R.H.); ujang.hidayat@unpad.ac.id (U.H.T.); 2 Vocational School, Universitas Sebelas Maret, Surakarta 57126, Central Java, Indonesia; 3 Department of Animal Nutrition and Feed Technology, Faculty of Animal Science, IPB University, Bogor 16680, West Java, Indonesia; anuragaja@apps.ipb.ac.id; 4 Department of Biology Education, Islamic University of Riau, Pekanbaru 28284, Riau, Indonesia; fitrirmdhani01@gmail.com; 5 Department of Animal Science, Politeknik Negeri Jember, Jember 68101, Jawa Timur, Indonesia; adib@polije.ac.id; 6 Research Center for Applied Zoology, National Research and Innovation Agency (BRIN), Jl. Raya Jakarta-Bogor Km 46, Cibinong, Bogor 16911, West Java, Indonesia; aini002@brin.go.id; 7 Research Center for Animal Husbandry, Research Organization for Agriculture and Food, National Research and Innovation Agency (BRIN), Jl. Raya Jakarta-Bogor Km 46, Cibinong, Bogor 16911, West Java, Indonesia; hidayat_c2p@yahoo.com; 8 INRAE, VetAgro Sup, UMRH, Université Clermont Auvergne, 63122 Saint-Genès-Champanelle, France; vincent.niderkorn@inrae.fr; 9 Department of Animal Nutrition, Faculty of Veterinary Medicine and Animal Science, Poznan University of Life Sciences, Wolynska 33, 60637 Poznan, Poland; adam.cieslak@up.poznan.pl (A.C.); malgorzata.szumacher@up.poznan.pl (M.S.-S.)

**Keywords:** saponin extract, ruminant, methane, performance, milk, N utilization, meta-analysis

## Abstract

**Simple Summary:**

The present meta-analysis was conducted to investigate the use of saponin extracts as dietary supplements for ruminants, based on in vivo studies. This study aimed to highlight the benefits of saponin extracts and their impact on ruminant health and production. A total of 26 articles, comprising 66 studies, were included in the database. The meta-analysis was designed to elucidate the effects of saponin extracts from different sources on production performance, milk yield, digestibility, rumen fermentation, nitrogen utilization, and blood metabolites. The results revealed that increased saponin supplementation linearly decreased milk production and altered rumen fermentation profiles without affecting digestibility rates, particularly influencing volatile fatty acid production and protozoa population. However, the efficacy and safety of different levels of saponin extracts vary, and further research is required to optimize its use for enhancing ruminant productivity, mitigating environmental impacts, and exploring the specific effects of saponin extracts on ruminant health. It can be concluded that the utilization of saponin extracts in ruminant diets is complex, and a comprehensive understanding of the optimal application of various saponin extracts across different ruminant physiological conditions is necessary.

**Abstract:**

The present meta-analysis aimed to determine the underlying effects of different saponins extracted from different sources on the production performance, milk yield, digestibility, rumen fermentation, blood metabolites, and nitrogen utilization of ruminants. A total of 26 papers comprising 66 in vivo studies (148 data points of dietary treatments) were evaluated in the present study. The databases were statistically analyzed using the mixed model procedure of SAS, where experiments considered random effects and tannin-related factors were treated as fixed effects. Statistical procedures were then continued in comparing different sources of saponin extract through Mixed Model analysis, where experiments were also random factors and sources of saponin extract were fixed factors. The evidence revealed in the present meta-analysis that saponin supplementation of up to 40 g/kg DM appears to have no detrimental impact on feed intake across ruminant types, suggesting that it does not significantly affect diet palatability. However, the results indicated that there are species-specific responses to saponin supplementation, particularly in relation to palatability and nutrient absorption efficiency, with larger ruminants being better able to tolerate the bitterness induced by saponin extracts. Furthermore, the study found that saponin extracts can influence nutrient digestibility and rumen fermentation dynamics, with different effects observed in large and small ruminants. While some saponin extracts can enhance average daily weight gain and milk yield, others can have adverse effects, highlighting the importance of considering both saponin sources and animal physiological condition when developing nutritional strategies. Additionally, optimization of ruminant production by utilizing saponin extracts is necessary to avoid negative health implications, such as increased blood creatinine levels. Different saponin extracts utilization in ruminant nutrition and environmental management, have a distinct understanding associated to their various bioactive properties. However, among the saponin sources, saponin extracted from *Quilaja saponaria* is more likely to improve large ruminant production performance while maintaining ruminant health and metabolism, but negatively affect small ruminants. Further research is needed to unravel the intricate effects of different saponin sources on ruminant health and productivity, emphasizing the importance of tailored dietary strategies that consider the unique physiological and metabolic characteristics of the target livestock.

## 1. Introduction

Ruminant nutrition has evolved its interest in utilizing natural plant compounds to sustainably produce good quality products of ruminant origin, such as meat and milk, effectively and efficiently, as well as maintaining animal health. One of the generally known natural plant compounds is saponins, which have recently emerged as a significant concern because of their beneficial mode of action in ruminant production [1]. Saponins are glycosides characterized by sugar and non-sugar bonds (such as aglycone or sapogenin) that have a soap-like mode of action. They are naturally found in a wide variety of plants, such as legumes and medicinal herb plants, at various concentrations because of their anti-nutritional factors that interfere with digestive metabolism [2]. In recent years, saponins have been introduced as effective natural compound agents to modulate the rumen fermentation and digestibility in ruminant [3]. 

Ruminant feed supplemented with saponins has demonstrated various beneficial effects, including promoting health and immune metabolism, modulating ruminal fermentation and digestibility, and mitigating methane production, which contributes to reducing the environmental impact of GHG emissions [4,5]. Extensive research has revealed that saponins possess potent antimicrobial properties that directly decrease the population of microorganisms, such as bacteria, protozoa, and methanogens, which are linked to a reduction in enteric methane production [6,7,8]. However, evidence regarding the beneficial effects of saponin extracts as health-promoting agents and their performance in ruminants is inconclusive [9]. For instance, a study revealed that saponins extracted from *Yucca schidigera* fed to dairy cattle at about 25–50 g/d had lower ruminal VFA concentration [10], but showed no effects on VFA concentration when saponin from the whole part of *Terminalia chebula* Retz. was introduced to goats [11]. Moreover, several studies have confirmed that saponins whether in a whole part sources of saponin or extracted form influence ruminal N-ammonia concentrations [12,13] and have detrimental effects on excreted N in feces and urine [13,14]. 

Inconsistencies regarding the effects of saponin supplementation were also found in performance parameter analyses, such as average daily gain, milk yield, N-utilization and ruminal digestibility [14,15,16]. Therefore, there is a need for extended evaluation of saponin utilization in ruminants, especially in extracted forms originating from various plant sources. Different plants containing saponin compound extracts have different efficacies in modulating ruminal fermentation in various ruminant species as well as their influence on animal performance and ruminal metabolism. Preeminently, saponin extract utilization in ruminant nutrition attracts interest, which can be related to the type of animals, dosage levels, and plant sources. Hence, the present me-ta-analysis aimed to determine the effects of saponin extract supplementation at various levels and sources (types) on production performance, milk yield, digestibility, ruminal fermentation, blood metabolites, and nitrogen utilization of ruminants.

## 2. Materials and Methods

### 2.1. Database Development

**Figure 1:**
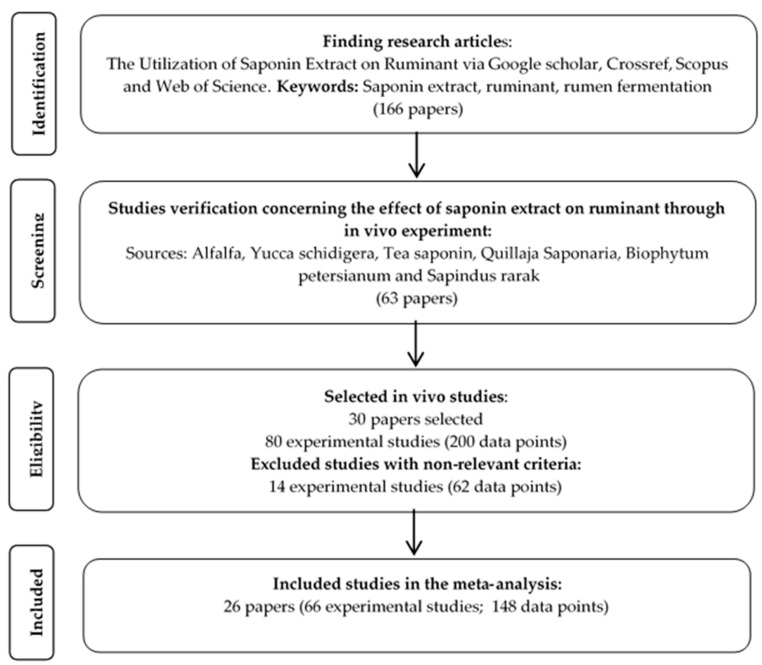
Diagram flow for selection of the studies on the influence of saponin extract.

The present study database was constructed from various studies which reported the nutritional utilization of saponins in extracted form. The data included in the constructed database were based on published articles written in English describing in vivo experiments. All included in vivo data were obtained from journals indexed in Google scholar, Crossref, Scopus and Web of Science such as graphically described in Figure 1.

The final database was set up from 26 experimental studies consisting of 66 dietary treatments (148 data points; Table 1). The criteria for incorporating articles into the database were as follows: (a) detailed description of in vivo experiments conducted on ruminants; (b) inclusion of extracted saponins into basal feeds within the studies; (c) consists information of observed variables such as dietary intake, average daily gain (ADG), milk yield and nutrient profile, total digestibility, ruminal fermentation characteristics, nitrogen utilization, and blood parameters; and (d) articles are written in English. In the present study, the source of saponins was determined from the extracts of alfalfa or *Medicago sativa* (MS), *Quillaja Saponaria* (QS), tea saponin or *Camelia sinensis* (CS), *Yucca schidigera* (YS), *Agave americana* (AA), *Biophytum petersianum* (BP), and *Sapindus rarak* (SR). Most studies have used a mixed basal diet composed of corn silage, wheat straw, canola meal, barley concentrate, elephant grass, wheat pollard, maize silage, ensiled brewer’s grains, ensiled beet pulp, meadow hay, rapeseed meal, and hay pelleted concentrate. Type of ruminants that were included in the database by the manner of saponin extract supplementation were steers, dairy cows and buffalo, categorized as type of large ruminants, while sheep, lamb, and goats, were categorized as type of small ruminants.

The saponin extracts supplementation units were standardized and presented as g/kg dry matter (DM) of feed. Moreover, the units of each measured value in the database for each parameter were standardized. Hence, all measured values in an observed parameter expressed in other than the common units were converted to get similar units. For instance, dry matter intake (DMI), organic matter intake (OMI), and neutral detergent fibre intake (NDFI) that are presented as g/d or kg/d were converted and expressed as g/kg metabolic body weight unit (g/kg BW^0.75^). The average daily gain (ADG) expressed as grams per day (g/d) and kilograms per day (kg/d) were converted into g/kg BW^0.75^ unit, while milk yield units reported as g/d and kg/d were converted and expressed as g/kg BW^0.75^ and g/kg DMI. Such unit conversion relative to metabolic body weight is necessary to reduce the variability of presented data by considering the type, and weight of trialed animals across studies. Similarly, N utilization units expressed as g/d and kg/d were also converted into g/kg BW^0.75^ unit. Moreover, digestibility rate, milk nutrient composition, milk nitrogen utilization, and VFA proportion units were standardized and presented as g/100 g or percentages. Additionally, ruminal fermentation profiles expressed in total VFA concentration, ruminal ammonia concentration, and blood plasma parameters were converted and presented in mmol/L, µmol/L, g/dL or mg/dL. Meanwhile overall data of protozoa population expressed in 10^x^/mL were converted into log_10_/mL unit.

### 2.2. Statistical Analysis

The statistical analysis was performed using a similar approach to the previous meta-analysis using SAS version 9.4 [17,18]. The raw dataset was assessed for outliers using the PROC REG of SAS and a minimum sample size of at least three studies was included in the analysis. To examine the effects of saponin extracts, we initially performed a meta-regression analysis by considering the inclusion levels of saponin extracts in the diets, type of animals, sources of saponins, and other relevant covariates that might have affected the observed parameters. Then, a categorical meta-analysis was performed followed Yanza et al. [19] and Respati et al. [18] statistical methods with some modification to explore the specific effects of saponin sources and their interactions with other factors in the dataset. For the meta-regression analysis, multiple models based on linear mixed models (LMM) were tested using the following model:

∆Υ*_ij_* = β_0_ + β_1_X*_ij_* + β_2_X*_ij_*^2^ + (β_1_ × β_3_…n)X*_ij_* × S*_i_* + ε*_ij_*, [full model]

∆Υ*_ij_* = β_0_ + β_1_X*_ij_* + β_2_X*_ij_*^2^ + (β_1_ × β_3_…n − 1)X*_ij_* × S*_i_* + ε*_ij_*, [reduced model]

where ∆Υ*_ij_* = estimated outcome of the dependent variable based on *j* observation in *i* experiment, β_0_ = estimated intercept (fixed effect), β_1_ = linear model coefficient of continuous predictor (fixed effect), β_2_ = quadratic term coefficient of continuous predictor (fixed effect), X*_ij_* = saponin extracts’ levels of *j* observation in *i* experiment, the matrix of the continuous predictor variable, β_3_ … β_n_ = coefficient of the categorical variables, S*_i_* = the random effect of studies, and ε*_i_* = the residual error at ~N(0,σ^2^).

The study employed a rigorous methodology to find accurate model. For instance, the CLASS statement was utilized to analyze varying concentrations of saponin extract supplementation and study variables without quantitative data, while the RANDOM statement was declared based on different experiments across studies. The models used weighing factors to represent the characteristic variability of each study, with the weights divided by the mean of all weights relatively to the number of observed variables of each level, as suggested by St-Pierre [20]. This methodology ensured that the results can maintain the expressions of dispersion in the original scale of the measurements and expressed robust, accurate, and reliable results.

A backward elimination procedure was followed to obtain the best-fitted model using Akaike’s information criterion (AIC), root-means square errors (RMSE), and between-models F-test of presented results. The linear model was retained if quadratic effect and other interaction effects were not statistically significant (*p* < 0.05) or tend to significant (0.10< *p* < 0.05). Then, the categorical meta-analysis was performed refered to Yanza et al. [21] protocol to examine the effects of the sources of saponins using the following statistical model:

Y*_ij_* = µ + β_a_ + (β_a_ × β_b_)x*_ij_* + sβ*_ij_* + S*_i_* + e*_ij_*

where Y*_ij_* = the estimated means of response variable Y of *j* observation in *i* study, µ = overall mean, β_a_ = fixed effect of categorical variables, β_b_ = fixed effect of the covariates, β_a_ × β_b_ = interaction terms between categorical variables and covariates of *j* observation in *i* study, sβ*_ij_* = random term between *i* study and the *j* factors β, S*_i_* = random term of the study, and e*_ij_* = residual error ~N(0,σ^2^). The effects were deemed significant at *p* < 0.05 and tended to significant at *p*-value between 0.05 and 0.10 using Tukey-Kramer’s test.

**Table 1:** Lists of studies included in the present meta-analysis study.

No	Study	Year	Exp.	Animal	Age (Months)	Status	IBW (kg)	Sources of Saponin Extract	Level (g/kg DM)	Extracted Forms
1	Valdez et al. [22]	1986	1	Dairy cows	n.d.	1st lactation (6–10 week postpartum)	n.d.	*Yucca schidigera*	0.77	Powder
2	Lu et al. [23]	1987	2–3	Sheep	n.d.	Mature wethers	49	*Medicago sativa*	20–40	Powder
3	Wu et al. [24]	1994	4–5	Dairy cows	n.d.	Lactation	650	*Yucca schidigera*	0–0.396	Powder
4	Hussain [13]	1995	6–15	Steers	n.d.	n.d.	574–658	*Yucca schidigera*	0.25	Powder
5	Wilson [25]	1998	16–17	Dairy cows		Multiparous (122 d postpartum)	640	*Yucca schidigera*	0.378	Powder
6	Sliwinski [26]	2002	18	Sheep	4.02	Castrated male lambs	35.1	*Yucca schidigera*	0.002–0.03	Powder
7	Eryavuz abd Dehoroti [8]	2004	19–22	Sheep	24–108	n.d.	186.6	*Yucca schidigera*	5–30	Liquid
8	Santoso et al. [27]	2006	23	Goat	n.d.	n.d.	20.3	*Biophytum petersianum*	0.072–0.144	Liquid
9	Wina et al. [28]	2006	24–25	Sheep	n.d.	Male sheep	16.5	*Sapindus rarak*	20.16–30.24	Powder
10	Lovett et al. [29]	2006	26–27	Dairy cows	n.d.	1st and 2nd or 3rd lactation (±39 d post calving)	585–610	*Yucca schidigera*	1.488–4.421	Powder
11	Baah et al. [3]	2007	28	Dairy cows	n.d.	Heifers	601	*Quillaja saponaria*	0–8	Powder
12	Liu et al. [30]	2007	29–34	Sheep	n.d.	Male sheep	40	*Yucca schidigera*	0.1–0.3	Powder
13	Abdelmawla [1]	2008	35	Buffalo	n.d.	4th and 5th lactation	591	*Quillaja saponaria*	0.052–0.052	Liquid
14	Benchaar et al. [6]	2008	36	Dairy cows	n.d.	Lactation (87 DIM)	730	*Yucca schidigera*	2.752	Powder
15	Singer et al. [31]	2008	37	Dairy cows	n.d.	Late lactation (298 DIM)	810	*Yucca schidigera*	2.01–6.23	Powder
16	Selcuk & Tuncer [32]	2010	38	Sheep	2–2.5	Male lamb	20.87–21.69	*Yucca schidigera*	0.2–0.4	Powder
17	Li et al. [16]	2011	39	Sheep	n.d.	Male sheep	40	*Yucca schidigera*	0.1–0.3	Powder
18	Nasri et al. [33]	2011	40–43	Sheep	5–6	Male lamb	17.8–18.8	*Quillaja saponaria*	0.1–0.09	Powder
19	Nasri et al. [34]	2012	44–55	Sheep	5–6	Female lamb	23.9–28.9	*Quillaja saponaria*	0.12–0.36	Liquid
								*Agave americana*	0.12–0.36	Powder
20	Guyader et al. [9]	2015	56–57	Dairy cows	n.d.	Multiparous nonlactating	658	*Camellia sinensis*	5	Powder
21	Guyader et al. [10]	2017	58	Dairy cows		Primiparous &	617	*Camellia sinensis*	7.6	Powder
						Multiparous (106 DIM)				
22	Baheg et al. [4]	2017	59–60	Sheep	43	Ewes	33.76	*Yucca schidigera*	0.2	Powder
23	Kumar et al. [14]	2017	61–63	Goat	7.03	Male kids	19.43–19.96	*Camellia sinensis*	4	Powder
25	Zhang et al. [35]	2021	65	Sheep	12	Male castrated sheep	48.37	*Camellia sinensis*	5–20	Powder
24	Yi et al. [36]	2022	64	Steers	n.d.	Steers	510.5	*Yucca schidigera*	0.198	Powder
26	Alsubait et al. [2]	2023	66	Sheep	3 and 4	Male lambs	26.26–26.97	*Yucca schidigera*	0.3–0.6	Powder

Exp. = number of experiments in the study; DIM = days in milk; IBW = initial body weight; DM = dried matter; n.d. = not determined.

## 3. Results

### 3.1. Datasets

The literature search in the present meta-analysis included 26 studies investigating the inclusion of saponin extracts supplemented in the diets of ruminants, together with information such as animal status, initial body weight during the experiment, source of saponin extract, and the range of saponin extract supplementation levels in the animal diet (Table 1). The present study observed that studies examining extracted saponins were dominated by *Yucca schidigera* and *Quillaja Saponaria* extracts. Information regarding the descriptive statistics of the datasets is presented in Table 2. The descriptive data also showed that, overall, the ranges of values of the observed parameters were within the expected values, although high variability within the studies was also identified by standard errors of the means (SEM).

### 3.2. Relationship between Dietary Saponins Levels on Observed Parameters

**Figure 2:**
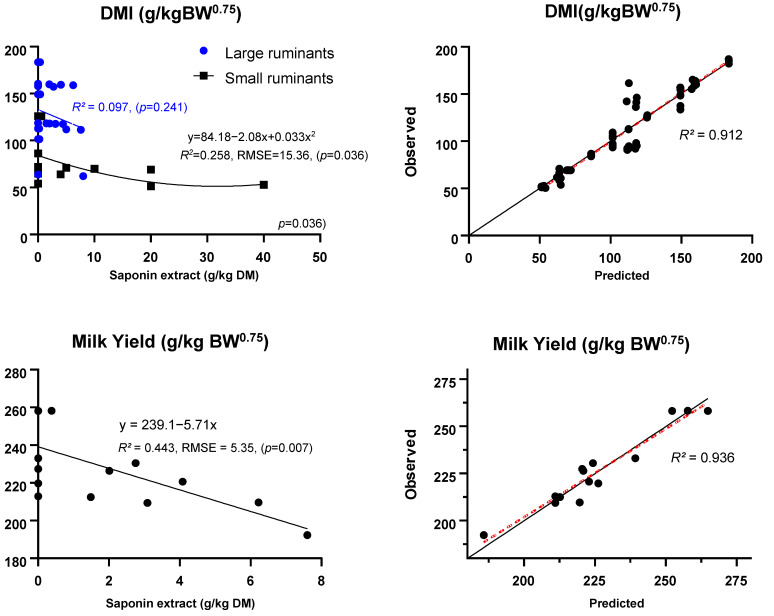
Meta-regression results representing the relationships between dietary saponin sources’ intake (g/kg DM) on dry matter intake and milk yield per metabolic body weight (BW^0.75^). The right figures represent the model performance evaluation according to the observed vs. predicted values.

Meta-regression indicated that the levels of saponins in the diets had no significant effect on DMI, OMI, and NDFI expressed as g/kg BW^0.75^ (Table 3). However, the interaction between the level of saponin extract and the type of animal was significant for DMI and NDFI (*p* < 0.05), but tended to be significant for OMI (*p* = 0.079). In Figure 2, the pattern of DMI (g/kg BW^0.75^) only showed a significant model for small ruminants in a quadratic manner (*p* = 0.036; *R*^2^ = 0.258), where high accuracy on the predicted models was adjusted (*R*^2^ = 0.912). Moreover, an interaction between the level of saponin extract and the type of saponin source was also observed for NDFI and OMI (*p* < 0.05).

The ADG expressed as g/d and g/kg BW^0.75^, was unaffected by the increased level of saponin extract. Nonetheless, the ADG expressed as g/kg BW^0.75^ showed a significant interaction between the level of saponin extract and the type of animals (*p* = 0.009), in which, results only represented by small ruminants. On the other hand, the meta-regression of milk yield expressed on kg/d and g/kg BW^0.75^ was linearly decreased (*p* < 0.005) with increasing levels of saponin extract supplementation. Moreover, supplementation with saponin extract and type of animal showed an interaction effect on milk yield expressed as kg/d, g/kg BW^0.75^, and g/kg DMI (*p* < 0.05), but the interaction between the level and type of saponin extracts was shown only when milk yield was expressed as kg/d (*p* = 0.01). The regression model in Figure 2 also confirmed that the milk yield, expressed as g/kg BW^0.75^, linearly decreased with the increased level of saponin extracts (*p* = 0.007, *R*^2^ = 0.443), with high accuracy of adjusted determination of the predicted model (*R*^2^ = 0.936). Increasing levels of saponin extract in the diet did not affect milk fat, milk protein, or milk lactose proportion in dairy ruminants. However, the interaction between the level of saponin extract on the type of animals and the type of saponin source was shown in milk fat proportion (*p* < 0.05). In contrast, the milk protein proportion showed a significant interaction between the level of saponin extract and the type of animals (*p* < 0.01). However, it tended to be significant when interacting with the type of saponin source (*p* = 0.082).

Interestingly, dry matter digestibility (DMD) showed a linear increase in response to the increased levels of saponin extract (Table 3; *p* = 0.048) respected with the interaction on the type of animal (*p* = 0.05), although the crude protein digestibility (CPD) was unaffected. No effects were shown by the increased level of saponin extract on organic matter digestibility (OMD), although it showed an interaction with animal type. Nevertheless, the present results also revealed a significant decrease (*p* = 0.036) in acid detergent fiber digestibility (ADFD), followed by an interaction with animal type (*p* = 0.05). However, there was no significant effect of the increased level of saponin extract on neutral detergent fiber digestibility (NDFD), although it showed a tendency to significantly interact with animal type (*p* = 0.064).

The inclusion of saponin extract appeared to influence ruminal fermentation parameters on total volatile fatty acid (VFA) production and individual VFA composition, followed by interaction with animal type (*p* < 0.05) and saponin source type (*p* < 0.001), except for pH and ammonia concentration that showed no significant effects. Moreover, the ruminal protozoa population significantly increased saponin extract levels in a quadratic pattern, including a significant interaction effect on saponin sources (*p* < 0.001).

The absence of effects of saponin extract supplementation on fecal N and N retention was also observed, but fecal N showed an interaction with animal type (*p* = 0.009). Meanwhile, Urine N tended to decrease with increasing levels of saponin extract supplementation (*p* = 0.057), with no interaction. Blood plasma biochemical parameters, including total protein, albumin, globulin, plasma urea nitrogen, cholesterol, glucose, and alkaline phosphatase (ALP), did not affect the increased level of saponin extract supplementation. However, cholesterol, glucose, and ALP tended to interact with type saponin sources (0.05 < *p* < 0.10). Nonetheless, blood creatinine concentration was increased in a quadratic manner (*p* = 0.014), followed by an interaction with the type of saponin source (*p* < 0.001).

### 3.3. Comparative Analysis of Saponins’ Sources

**Figure 3:**
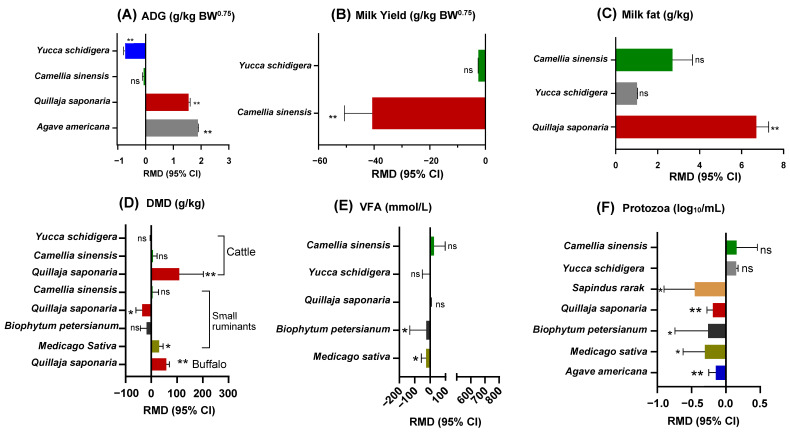
The effects of different sources of saponin extracts on average daily gain, dry matter intake, total volatile fatty acids production, and rumen protozoa population in ruminants. Results are presented as raw mean difference at 95% confidence intervals with control diets as a comparator. *: *p* < 0.05; **: *p* < 0.01; ns: no significance.

The comparative effects of different saponin sources on ruminants are presented in Table 4 and Figure 1. The results showed that the inclusion of various saponin extracts in the diets did not affect (*p* > 0.05) DMI, OMI, and NDFI but showed significant differences in the interaction between the type of saponin extract and the type of animals (*p* < 0.05). Moreover, different effects on ADG were observed; both the AA and QS extracts had relatively higher ADG, and the YS extract was relatively lower compared to the control group (CON). As displayed in Figure 3, dietary inclusion of YS extract reduced (*p* < 0.05) the ADG of ruminants. At the same time, QS and AA favorably increased (*p* < 0.05) the ADG of ruminants, where the ADG parameter in the database was only shown for the small ruminant type.

The effects of CS extract were consistent in dairy animals, whereas CS decreased (*p* < 0.05) milk yield (expressed as kg/d and g/kg BW^0.75^ units (*p* < 0.05; Figure 3B). Moreover, a higher (*p* < 0.05) milk fat proportion was observed in the QS extract group than in the CON group (*p* < 0.001; Figure 3C). In general, there were no effects of various saponin sources on digestibility parameters compared to CON, but when they showed an interaction between sources and type of animals, DMD and OMD were significant (*p* < 0.05). For example, in Figure 3D, there were showed that the digestibility among different types of ruminants showed an increased DMD on QS for large ruminants, i.e., buffalo and cattle, compared to the CON group (*p* < 0.05). However, in small ruminants, the supplementation of QS lowered DMD compared to the CON group, whereas MS had a higher DMD than the control (*p* < 0.05).

Regarding rumen fermentation parameters, only supplementation with MS and BP extracts resulted in a lower total VFA concentration than the CON group (*p* < 0.01). Nonetheless, supplementation with MS, QS, AA, BP, and SR extracts (*p* < 0.05) significantly diminished ruminal protozoa population, except for the CS and YS extracts. No effects of various types of saponin extract on N partitioning parameters were observed, except for the interaction of sources with animal types on Fecal N (*p* = 0.001). However, the AA extract had higher PUN and creatinine concentrations in the blood than the CON group (*p* < 0.001). Moreover, QS and AA extracts significantly reduced blood glucose concentration in blood than the CON (*p* = 0.005).

## 4. Discussion

Many studies have been conducted over the past few decades to investigate the potential benefits and drawbacks of saponins on the health and productivity of ruminants. Researchers have primarily focused on identifying natural sources of saponins and observing their positive effects on livestock health, production performance, and enteric methane emissions from ruminants [9,10,24]. Most studies have widely recognized the use of saponins in ruminants, either in their whole plant form or as an extracted defaunation agent, which can improve nutrient utilization efficiency and consequently affect production and product quality, as well as environmental impacts such as methane emissions [37,38,39]. However, the current study’s hypothesis is focused on systematically determining the influence of saponin utilization in the extracted form, which consists of pure saponin compounds rather than the whole plant parts that considered as a source of saponins. The effects of saponin extract levels on observed parameters were analyzed in a meta-regression analysis, which was then compared to the type of saponin extracts. The results revealed the association between the levels of saponin extract and the type of animals or sources of saponin extract on the observed parameters. A comparative meta-analysis was performed to determine the association between the type of saponin extract and the influencing parameters in ruminants.

### 4.1. Influence of Saponin Extract Utilization on Ruminant Performance, Digestibility, Rumen Fermentation and Health Parameters

The current study revealed that various levels of saponin extract up to 40 g/kg DM did not negatively affect feed intake, indicating that the palatability of the diets was generally unaffected. However, it was found that the type of animal and the dietary intake of saponin extract interacted, which led to an increased interest in the differences in feed intake between large and small ruminants. Additionally, factors such as the weight and size of ruminants can affect the efficiency of nutrient uptake from diets containing saponin extracts. The quadratic regression model in Figure 2 shows that small ruminants had a reduced palatability pattern (DMI, g/kg BW^0.75^) due to an increased level of dietary saponin extract (g/kg DM; *p* < 0.036), whereas there was no significant effect on larger ruminants. Some studies have suggested that saponins may decrease feed intake owing to their bitter taste [40,41]. Therefore, it is suggested that large ruminants have a more diverse palatability to tolerate the bitter taste of saponins than small ruminants [9,42]. This evidence indicates that large ruminants may naturally mitigate the adverse effects of saponin extract and have more efficient energy utilization when fed diets containing saponin extract [43].

Moreover, the interaction effects between the level of saponin extract with extract sources and animal types on nutrient digestibility were also reported in this study. DMD and OMD rates were increased (*p* < 0.05), while adverse effects were observed on NDFD and ADFD rates, where saponin extract levels interacted with ruminant type (0.05 < *p* < 0.10 and *p* = 0.005, respectively). These findings show the complexity of the noticeable influence of saponin extract mode of action that is associated with ruminant types, rumen microbiota, and digestion kinetics, where large ruminants possess a larger rumen volume that may extend their environment for microbial activity, although the bioactivity of saponin might also influence the fermentation process [38,43,44]. Furthermore, impaired digestibility might further enforce performance production in ruminants.

Despite the fact that the application of dietary saponin extract at various levels did not produce any discernible effect, the average daily weight gain (ADG) of ruminants that consumed saponin at different levels was found to be influenced by both the type of animal and the source of saponin extract. The results revealed that there was a significant interaction between these two factors, with the type of animals expressed as g/d (*p* = 0.021) and the type of saponin extract source expressed as g/kg BW^0.75^ (*p* = 0.009). However, the current findings cannot be relied upon to draw definitive conclusions regarding the effect of increased saponin extract levels on daily weight gain, as the available evidence may be limited. The ADG data in the current study were collected only for small ruminants, and the inclusion of saponin extract in the meta-regression model negatively affected their natural metabolism. These findings underscore the potential differential bioactivity of saponin compounds, which may depend on the physiological characteristics of ruminants, as previously reported in the literature [9,44].

Decreased milk yield and altered composition indicate a complex interplay of factors during ruminal fermentation and nutrient absorption, which is influenced by the mode of action of the saponin extract (*p* < 0.01). The effect of various saponin extract levels on milk yield, expressed as g/kg BW^0.75^, was evident, with a linear decrease observed as saponin extract supplementation increased in the diet (*p* < 0.01; Figure 2). Although the influence of supplementation levels and different types of saponin extracts on ruminants may vary, the antimicrobial activity of saponins against certain protozoa and bacteria could result in a decrease in volatile fatty acid (VFA) synthesis, which is essential for the metabolizable energy required for dairy ruminants to produce milk [33,34,45]. In the present study, the total VFA concentration, as well as the proportion of acetates and valerates, decreased due to increased saponin extract levels (*p* < 0.05). However, the interaction between levels and source saponin extract, as well as between levels and type of animals, was confirmed for milk fat (*p* < 0.01) and protein proportion (*p* < 0.10). Some studies have demonstrated the effects of saponins on rumen microbial activity by inhibiting the lipid biohydrogenation (BH) process in the rumen [10,46]. Therefore, inhibiting the ruminal BH process of long fatty acids (FA) may further enhance the increased fat proportion in milk.

The link between increased saponin extract supplementation and altered ruminal fermentation products has been well documented in previous research. In these studies, the majority of saponins have demonstrated antiprotozoal and methanogenic effects [17,28,34]. However, a decrease in VFA and acetate proportion could be related to a reduction in fiber-degrading microorganisms, such as cellulolytic bacteria and rumen protozoa, which play a critical role in fiber degradation [36]. Currently there have been noticed that *Ruminococcus* sp. and *Bacillota* bacteria genera are responsible for the degradation of cellulose and hemicellulose [36]. On the other hand, other studies also assured that rumen protozoa produce enzymes essential for the breakdown of complex carbohydrates in plant material; thus, the diversity and abundance of these enzymes contribute to the breakdown and fermentation of fiber [47]. Additionally, previous studies have found that dietary saponins improve nutrient digestibility, particularly of fiber, by selectively inhibiting protozoa, which the increased the growth of fiber-degrading bacteria [47]. As a result, the degradation rate of feed particles in the rumen is influenced by a decrease in certain protozoan families. However, the present study did not analyze rumen bacterial activity because of the limited number of studies on saponin extract supplementation. Despite this, the impact of the antimicrobial activity of saponin extract on milk production can be explained by the role of digested nutrient metabolic pathways, which influence nutrient deposition in mammary glands [48].

The population of protozoa increased quadratically with saponin extract supplementation, with some types exerting an interaction effect (*p* < 0.05 and *p* < 0.01, respectively). Previous studies have shown that saponin can increase rumen microflora, such as cellulolytic bacteria, and inhibit protozoan activity, with effects closely related to increased ammonia and VFA modulation in the rumen [17,36]. However, the results of the present study contradict these findings. Different saponin sources may have varying effects on microbial populations. For example, *Sapindus rarak* and *Quilaja saponaria* extracts effectively reduced protozoa and bacterial numbers [28,35], whereas certain levels of *Yucca schidigera* and *Camelia sinensis* increased protozoa and bacterial populations [8,9]. These findings highlight the different susceptibility rates of direct and indirect effects of various types and levels of saponin extracts on ruminal microbes [28,48,49]. These inconsistencies in microbial populations could be due to the type of saponin source, dietary ration, and duration of supplementation [36]. Additionally, reduced ruminal microbial activity, which is often associated with increased milk production and metabolism, is linked to reduced enteric methane production [50]. However, the present meta-analysis did not provide substantial evidence regarding the influence of different types and levels of saponin extract supplementation on enteric methane emissions from ruminants in vivo. Therefore, further research is needed to investigate the long-term effects of saponin extracts from different sources or at various levels, and to determine their direct impact on enteric methane mitigation in ruminants.

The results of this study suggest that the saponin extract has a modulatory effect on the metabolic health of ruminants. The levels of urinary N excretion tended to decrease according to the quadratic model (*p* = 0.08), with no significant changes in N retention or fecal N. However, the effect of saponin extract on the N cycle could not be determined. These findings are supported by Wina et al. [28], who found that ruminal protozoa defaunated by *Sapindus rarak* extract supplementation resulted in decreased protein degradation. Tea saponin extract was also confirmed to lower ammonia levels and thus reduce urinary-N excretion. Hence, the effect of saponin extract might attributed to its ability to diminished protozoa whereas consequently enhance ruminal fiber-degrading bacteria, and increased microbial protein synthesis. Available dietary nitrogen then is captured by microbial biomass and passed to the intestine for absorption, rather than being lost as ammonia and excreted as urinary-N and fecal N [51].

In contrast, a higher blood creatinine concentration was identified in a quadratic model (*p* = 0.014) followed by the interaction model between levels and source of saponin extracts (*p* < 0.001). Additionally, there was a tendency for an interaction between levels and sources of saponin extracts in cholesterol, glucose, and alkaline phosphatase in blood plasma (0.05 < *p* < 0.10). It appears that the ruminal microbial activity in ruminants fed specific types of saponin extracts affects the post-ruminant nutrient absorption process, which further absorbs metabolizable nutrients that are also modulated in the blood. Lowering cholesterol and glucose levels and increasing creatinine in blood serum in ruminants fed with saponin extract might indicate favorable health conditions. Previous studies have confirmed that changes in blood serum parameters, such as decreases in serum urea, creatinine, cholesterol, and liver enzyme activities, suggest that saponin’s anti-inflammatory and antioxidant modes of action could positively affect kidney function and metabolic health [1,52]. However, increased creatinine levels in the present meta-analysis might demonstrate the inconsistencies and high variability of experimental studies in ruminants fed with saponin extract. Hence, future studies may raise concerns regarding the effects of saponins on ruminant health and organ function, such as the kidneys. Previous clinical studies have confirmed that increased creatinine in the blood serum of living organisms might indicate kidney failure [53,54].

Supplementation with saponin extract has been associated with modifications in multiple performance parameters in ruminants, including feed intake, daily weight gain, milk yield, and milk nutrient composition. It has been observed that large and small ruminants exhibit distinct effects on milk yield and nutrient composition, particularly when supplemented with saponins [42,44]. Although the digestibility rates, ruminal fermentation effectivity, N utilization, and blood parameters between large and small ruminants also displayed intricacy, the present meta-analysis provides robust evidence regarding the effects of dietary saponin extracts on ruminant production, such as ADG and Milk yield, relative to ruminant metabolic weights (Figure 2). Moreover, a comparative analysis of different saponin extracts can elucidate the varying effects of each extract on the observed parameters in ruminants.

### 4.2. The Relationship between Ruminant Production Health and Metabolism by the Divergence Sources of Saponin Extract

The daily weight gain (ADG) of ruminants, expressed as g/kg BW^0.75^, significantly decreased when supplemented with YS extract, while positive effects were observed with QS and AA extracts (Figure 3A; *p* < 0.05) compared to the control (CON), which refers to small ruminants. Only three studies reported a positive effect of QS extract and AA on ADG parameters [3,33,34]. However, these corresponded to only one study that assessed the effect of QS extract on dairy buffaloes [1]. Similarly, only one study has reported the effect of QS extract on ADG parameters. Other studies have reported adverse effects of YS extract [2,32], whereas only one study reported that CS extract supplementation had no effect on ruminant daily weight gain [35]. Due to the small sample size, YS, QS, and AA extracts may not be statistically sufficient to provide general implications for observed ADG and thus warrant further investigation.

The results indicated that only the tea saponin extract (CS) significantly reduced milk yield, expressed as g/d and g/kg BW^0.75^ (Figure 3B; *p* = 0.001), compared to the control treatment. The strong bitter taste of the CS extract might impair ruminant palatability [35,38]. Similar to QS, CS contains triterpenoid saponins, a glycosides group that are well known for their remarkable bioactive diversity with multiple therapeutic benefits as anti-inflammatory, anti-microbial, and antioxidant properties [37]. In the studies using CS as the source of saponins, the results have been consistent to reduce milk yield [9,10,11,39]. Hence, the potent bioactivity of the CS extract could negatively affect rumen microflora, thereby long-term exposure of CS extract reducing the beneficial metabolizable energy to transporting nutrients [33,55] that are synthesized in milk concentrated in the mammary glands of dairy ruminants.

A meta-analysis by Yanza et al. [17] also confirmed that dietary CS extract (tea saponin) negatively affects ruminal protozoa and reduces digested dried matter, but positively influences VFA concentration and effectively mitigates methane emissions. In this study, digestibility was not reduced, indicating that the various saponin extracts had no negative impact on the degradation of fibrous nutrients. However, when digestibility (DMD) results were segregated based on ruminant type, QS extract was positively influenced in cattle and buffalo compared to the control (*p* < 0.001). Moreover, the QS extract negatively influenced small ruminants, whereas the MS extract positively affected DMD (*p* < 0.05). These findings suggest that only the MS extract (known as Lucerne or alfalfa) is susceptible to small ruminants, whereas the QS extract may have a detrimental effect, and further frequent uptake may lead to metabolic disorders. This evidence shows that the QS extract has stronger triterpene glucoside compounds than other saponin extract sources, which are toxic to small ruminants, especially when supplemented in a long term and at high levels [55]. However, large ruminants such as cattle and buffalo are more susceptible to saponin extract supplementation, and sophisticated results on digestibility have been obtained with QS extract supplementation. Therefore, different saponin extract sources are suitable only for specific types of livestock ruminants.

The results of this study indicate that supplementation with MS and AA extracts led to a reduction in rumen VFA concentrations compared to the control treatment (*p* < 0.001; Figure 3E). Although CS extract may positively influence the modulation of fiber-degrading bacteria, which could increase ruminal VFA concentration and fiber digestion, it may not be beneficial for improving lactating performance in dairy ruminants, specifically milk yield and its nutrient compounds. In contrast, the population of rumen protozoa was significantly decreased in response to MS, QS, AA BP, and SR extract supplementation (*p* < 0.01; Figure 3F), while no significant influence of CS, and YS extracts on ruminal protozoa population (Table 4). Correspondingly, although some sources of saponin extracts are well documented on exhibited a defaunating effect, the maximum protozoal reduction was only approximately 8% in this meta-analysis (SR vs. CON; 5.02 vs. 5.48 log10/mL; Table 4). Typically, negative effects on ruminal fermentation and nutrient digestibility occurred when the reduction of protozoal count was higher than 50%, as observed in previous in vitro studies using whole part of saponin sources levels as high as 40 g/kg DM [12,55], which was 10-fold higher than the inclusion levels in the studies we used. According to those studies, diets containing up to 0.4% of the whole part of saponin sources do not cause unfavorable effects on ruminal fermentation and nutrient digestion in ruminants.

The antimicrobial effect of saponin to protozoa has been described previously, because saponins form sterol-membrane complex damage and disintegrate the protozoa membrane and thus suppress the ruminal protozoa population [17,37]. However, the impact of CS extract on ruminal protozoa was inconsistent. The absence effect of CS extract on protozoal count, as reported by [10,11], coupled with the other studies reporting an increase in protozoal counts [9,17,41] suggested possible adaptation of ruminal protozoa against CS extract saponin. Perhaps, long-term exposure of ruminal protozoa to various saponin sources needs to be investigated. Generally, some previous study showed, that whole part saponin sources have been observed to have differing effects depending on their sources; however, their mode of action depends on their direct or indirect effect on the microorganisms involved in rumen fermentation [49,56]. Moreover, a high-forage diet for dairy cows containing high levels of feed like lucerne silage (source of saponin) may reduce protozoa population without negatively affecting the basic fermentation parameters and keeping milk production at a certain amount as in control group [50].

Only the QS extract supplementation resulted in a higher fat proportion in milk (*p* < 0.001). Although CS and YS extract might potentially increase fat proportion in milk but not significantly higher than the CON treatment (Figure 3F). This finding is aligned with previous studies reporting no effect of CS on milk fat and fatty acids profile [5,39]. This might be associated with the associated relationship between supplementary saponin extract with the increased activity of fiber degrading bacteria in producing acetate. Because acetate is known as the main precursor of milk fat biosynthesis [10]. Moreover, saponin mode of action might inhibit lipolytic bacteria on fatty acids BH process, hence, deposited essential unsaturated fatty acids may increase in milk fat [50]. Although no significant comparison compared to CON in the present results (Figure 3C), the positive effect of YC on milk fat synthesis has also been reported [29,31]. These evidences can be attributed to the effect of saponin extracts on modulating bacteria and protozoa activity those who responsible on producing VFAs and modulating fatty acids through BH process in the rumen [34,47,50]. Because the most noticeable effects can be seen from the decreased ciliate protozoal counts [23].

The administration of AA extract was associated with elevated levels of plasma urea-N and creatinine (*p* = 0.001). However, supplementation with QS or AA extract resulted in reduced blood glucose levels (*p* < 0.005). Elevated levels of urea and creatinine can be indicative of impaired kidney function or kidney disease, as the kidneys may not effectively filter these substances out of the blood [57]. Urea is a metabolic waste product that should be excreted in the urine, but the ability of AA extract to reduce protein synthesis can affect the levels of urea and creatinine in the bloodstream. The low levels of blood creatinine observed in ruminants supplemented with AA extract (1.22 µmol/L or 1.4 mg/dL) are still within the normal range (0.8 to 2.0 mg/dL) [58,59], suggesting no significant impact on kidney function.

Incorporating triterpenoid glycosides, such as AA and QS extracts, into the diet of ruminants may reduce the population of defaunated ruminal protozoa. These extracts form insoluble complexes, with hederagenin as the aglycone, that are effective in lowering blood glucose and cholesterol levels. Glucose is directly attached to hederagenin, while rhamnose and arabinose are linked to saponins in the rumen. These chemical linkages may be harmful to ruminal microbes, hindering the degradation and absorption of glucose and leading to changes in the bloodstream. The metabolism of plasma blood suggests that the bioactivity of saponin compounds can either promote or inhibit health conditions and growth performance, depending on the physiological characteristics of the animal. The effects of saponin extracts on ruminant health are dependent on the source of the saponins, as indicated by the results of the current study on the magnitude of the effects.

## 5. Conclusions

This meta-analysis found that the utilization of saponin extract in ruminants up to a certain threshold of 40 g/kg DM generally did not negatively impact feed intake and, therefore, did not compromise the palatability of ruminant diets. However, the type of ruminant and the level of saponin extract interaction suggests differential effects on nutrient absorption efficiency based on size. Specifically, small ruminants showed reduced palatability at higher saponin levels, indicating a species-specific tolerance threshold for the bitter taste associated with saponin extracts. This finding suggests that larger ruminants may possess adaptive mechanisms to mitigate the adverse taste effects of saponin extracts, potentially optimizing energy utilization from saponin extracts used in ruminant diets. The relationship between saponin extracts and various health and fermentation parameters is complex and shows that saponin supplementation has been linked to notable increases in average daily weight gain and milk yield, depending on the type of saponin extract and the animal’s physiological condition. However, certain saponin extracts have been shown to have adverse effects on milk yield and composition due to alterations in ruminal fermentation and nutrient absorption. The antimicrobial properties of saponins, which can reduce methane emissions and improve nutrient digestibility, can also disrupt the ruminal microbial ecosystem. This disruption can lead to a decrease in volatile fatty acid synthesis, which is essential for energy metabolism in dairy ruminants, ultimately influencing the production performance of dairy ruminants.

Some studies suggest that saponins can lower blood glucose and cholesterol levels, potentially indicating a positive shift in metabolic health, others have raised concerns about the potential impacts on kidney function. In particular, increased blood creatinine levels associated with certain saponin extracts, such as *Agave americana*, necessitate a cautious approach to dietary supplementation. The meta-analysis conducted in this study highlights the role of different saponin extracts in ruminant nutrition and environmental management, advocating for a distinct understanding of their various bioactive properties. However, among the saponin sources, saponin extracted from *Quilaja saponaria* is more likely to improve large ruminant production performance while maintaining ruminant health and metabolism, but negatively affect small ruminants. Further research is needed to unravel the intricate effects of different saponin sources on ruminant health and productivity, emphasizing the importance of tailored dietary strategies that consider the unique physiological and metabolic characteristics of the target livestock.

## Figures and Tables

**Table 2:** Descriptive statistics of the dataset used in the meta-analysis.

Parameters	Unit	N	Mean	SEM	Min	Max
Feed Intake						
DMI	g/kg BW^0.75^	67	113.1	4.917	50.49	187.0
OMI	g/kg BW^0.75^	12	80.25	9.159	48.82	150.2
NDFI	g/kg BW^0.75^	12	36.22	3.146	18.12	56.62
Gain Performance						
ADG	g/d	18	150.8	20.20	59.6	275.0
	g/kg BW^0.75^	18	13.18	2.209	5.25	27.80
Milk production and composition						
Milk yield	kg/d	21	25.23	2.071	7.01	33.85
	g/kg BW^0.75^	19	194.0	16.33	58.48	264.8
	g/kg DMI	21	1.20	0.080	0.46	1.55
Milk fat	g/kg	21	44.49	3.103	31.3	74.6
Milk protein	g/kg	17	34.22	1.358	28.0	47.9
Milk lactose	g/kg	12	47.56	0.785	43.8	52.2
Digestibility						
DMD	g/kg	40	698.3	8.769	628	813.0
OMD	g/kg	57	683.9	11.47	405	827.0
CPD	g/kg	44	636.2	10.46	531	793.7
NDFD	g/kg	47	559.0	14.89	295	730.3
ADFD	g/kg	27	489.9	15.79	323	660.1
Rumen fermentation parameters						
pH		148	6.30	0.030	5.51	7.1
NH_3_	mg/dL	146	18.77	1.028	4.12	81.74
Total VFA	mmol/L	74	103.7	14.21	48.9	125.0
Acetate	%	71	62.23	1.305	5.73	78.4
Propionate	%	71	22.52	0.835	3.14	41.74
Butyrate	%	71	11.65	0.432	1.17	19.3
Valerate	%	29	1.27	0.141	0.16	3.68
A:P ratio		71	3.10	0.151	0.79	7.47
Protozoa	log_10_/mL	91	5.42	0.044	4.19	6.52
N Balance						
Urine N/MBW	g/kg BW^0.75^	35	585.4	51.2	174.7	1099
Fecal N/MBW	g/kg BW^0.75^	35	550.2	46.5	199.8	1494
N retention/MBW	g/kg BW^0.75^	23	463.6	49.8	52.3	759.8
Blood parameters						
Plasma NH_3_	µg/dL	40	1.90	0.386	0.64	10.85
Total protein	g/dL	20	6.97	0.255	5.32	10.31
Albumin	g/dL	13	3.01	0.105	2.55	3.84
Globulin	g/dL	10	3.72	0.158	2.97	4.66
PUN	mg/dL	72	17.22	1.20	3.59	42.0
Cholesterol	g/dL	21	75.43	8.087	28.0	153.7
Creatinine	μmol/L	28	80.0	3.814	35.4	106.7
Glucose	mg/dL	20	55.17	3.524	30.96	86.0
ALP	IU/L	10	146.6	19.837	103.7	272.9

N = sample size; SEM = standard error of the mean; DMI = Dry Matter intake; OMI = Organic matter intake; NDFI = neutral detergent fiber intake; BW^0.75^ = metabolic body weight; ADG = Average Daily Gain; DMD = dry matter digestibility; OMD = Organic matter digestibility; CPD = Crude Protein digestibility; NDFD = neutral detergent fiber digestibility; ADFD = acid detergent fiber digestibility; VFA = volatile fatty acids; NH_3_ = ammonia; A:P = acetate to propionate ratio; PUN = Plasma urea-N; ALP = alkaline phosphatase.

**Table 3:** Results of meta-regression analysis of the effects of dietary saponin levels on ruminants.

Estimated Variables	Unit	N	Model	Intercept	SE_intercept_	Slope	SE_slope_	*p*-Value	RMSE	AIC	L × Animal	L × Source
Feed Intake												
DMI	g/kg BW^0.75^	67	L	112.1	7.822	−0.015	0.103	0.889	3.676	519.5	0.016	0.660
OMI	g/kg BW^0.75^	12	L	80.85	13.59	−0.216	0.725	0.778	4.728	97.8	0.079	0.005
NDFI	g/kg BW^0.75^	12	L	36.32	4.690	−0.036	0.288	0.905	1.878	77.98	0.032	0.021
Gain performance												
ADG	g/d	18	L	170.8	54.36	0.183	0.538	0.739	96.11	143	0.739	0.021
	g/kg BW^0.75^	18	L	12.01	4.198	0.009	0.061	0.880	0.705	83.1	0.009	0.854
Milk production and composition												
Milk yield	kg/d	21	L	25.98	3.932	−0.364	0.109	0.005	11.31	93.9	0.016	0.001
	g/kg BW^0.75^	19	L	211.6	29.18	−2.938	0.912	0.008	5.339	158.4	<0.001	0.295
	g/kg DMI	21	L	1.253	0.134	−0.002	0.005	0.698	0.030	−21.1	<0.001	0.220
Milk fat	g/kg	21	L	42.48	5.761	0.257	0.233	0.290	77.10	118	0.001	0.001
Milk protein	g/kg	17	L	33.70	2.485	−0.091	0.384	0.817	79.74	92.5	0.035	0.082
Milk lactose	g/kg	12	L	47.59	1.567	0.005	0.225	0.981	36.44	56	0.197	0.341
Digestibility												
DMD	g/kg	40	L	702.0	17.07	0.973	0.473	0.048	31.45	382	0.015	0.425
OMD	g/kg	57	L	687.2	26.60	0.373	0.371	0.320	59.26	543	0.002	0.359
CPD	g/kg	44	L	640.2	24.20	2.017	1.546	0.201	36.40	439	0.206	0.568
NDFD	g/kg	47	L	564.8	32.90	−1.345	0.806	0.104	48.43	471	0.064	0.795
ADFD	g/kg	27	L	500.7	33.10	−3.977	1.764	0.036	39.99	275	0.005	0.235
Rumen Fermentation Profile												
pH		148	L	6.301	0.068	−0.0004	0.005	0.945	0.176	114	0.793	0.394
NH_3_	mg/dL	146	L	23.11	3.102	−0.116	0.110	0.293	10.46	982	0.361	0.653
Total VFA	mmol/L	74	Q	107.9	25.50	18.44	6.010	0.003	78.17	897	<0.001	<0.001
						−0.440	0.150					
Acetate	%	71	Q	62.59	3.010	−0.640	0.320	0.049	6.880	494	0.001	<0.001
						0.010	0.008					
Propionate	%	71	L	21.17	1.848	−0.059	0.067	0.382	4.440	416	0.001	<0.001
Butyrate	%	71	L	11.28	0.943	−0.031	0.042	0.463	2.014	348	0.038	0.001
Valerate	%	29	Q	1.370	0.260	0.190	0.080	0.001	0.355	32.5	<0.001	0.001
			L			−0.040	0.010					
A:P ratio		71	L	3.321	0.358	0.002	0.008	0.826	0.796	140	0.949	0.786
Protozoa	log_10_/mL	91	Q	5.390	0.130	0.020	0.010	0.030	0.939	38.6	0.689	<0.001
						−0.001	0.000					
N Partitioning												
Urine N	g/kg BW^0.75^	35	Q	614.1	83.99	−5.524	3.315	0.057	38.02	430.9	0.371	0.934
						0.173	0.085					
Fecal N	g/kg BW^0.75^	35	L	573.96	70.65	−0.462	0.878	0.605	31.28	417.7	0.009	0.959
N retention	g/kg BW^0.75^	23	L	476.43	83.87	−0.914	2.207	0.686	40.00	272	-	0.976
Blood parameters												
Total protein	g/dL	20	L	6.790	0.498	−0.027	0.255	0.915	1.261	59.2	0.562	0.993
Albumin	g/dL	13	L	2.976	0.222	0.044	0.050	0.400	3.484	6.8	0.156	0.329
Globulin	g/dL	10	L	3.756	0.358	−0.057	0.096	0.576	7.530	15.7	0.243	0.226
PUN	mg/dL	72	L	18.23	2.317	0.105	0.559	0.852	1.426	378.5	0.156	0.535
Cholesterol	mg/dL	21	L	86.18	23.49	−1.849	3.145	0.565	94.65	160	0.679	0.055
Creatinine	μmol/L	28	Q	77.54	7.510	51.85	19.62	0.014	58.79	218	0.884	<0.001
						−13.37	4.960					
Glucose	mg/dL	20	L	49.85	8.310	−1.027	3.707	0.785	115.6	153	0.785	0.083
ALP	IU/L	10	L	166.8	37.75	0.514	2.830	0.861	98.59	76.9	0.861	0.065

DMI = Dry matter intake; OMI = Organic matter intake; NDFI = neutral detergent fiber intake; BW^0.75^ = metabolic body weight; ADG = Average Daily Gain; DMD = dry matter digestibility; OMD = Organic matter digestibility; CPD = Crude Protein digestibility; NDFD = neutral detergent fiber digestibility; ADFD = acid detergent fiber digestibility; NH_3_ = ammonia; VFA = volatile fatty acids; A:P ratio = acetate propionate ratio; N = nitrogen; PUN = Plasma urea nitrogen; ALP = alkaline phosphatase; L = linear term; Q = quadratic term; N = sample size; SE_intercept_ = standard errors of intercept; SE_slope_ = standard error of the slope; AIC = Akaike information of criterion; RMSE = root mean square error; L × Animal = interaction effects between levels of saponin extracts and type of animals; L × Source = interaction effects between levels and sources of saponin extracts.

**Table 4:** Results of meta-analysis based on the sources of saponins.

Estimated Variables	Unit	*n*	Experimental Groups								SEM	*p*-Value	Source × Animal
			CON	MS	QS	CS	YS	AA	BP	SR			
Feed Intake													
DMI	g/kg BW^0.75^	67	112.8	113.6	111.1	112.4	111.2	-	-	-	4.92	0.892	0.015
OMI	g/kg BW^0.75^	12	84.92	-	57.26	84.77	-	-	-	-	9.16	0.776	0.043
NDFI	g/kg BW^0.75^	12	39.53	-	18.54	39.99	-	-	-	-	3.15	0.142	0.017
Gain performance													
ADG	g/d	18	171.1 ^ab^	-	188.0 ^a^	169.9 ^ab^	164.8 ^b^	189.4 ^a^	-	-	44.43	0.005	-
	g/kg BW^0.75^	18	11.707 ^b^	-	13.25 ^a^	11.63 ^b^	10.97 ^c^	13.58 ^a^	-	-	2.21	0.025	-
Milk production and composition													
Milk yield	kg/d	21	25.80 ^a^	-	26.72 ^a^	20.81 ^b^	25.55 ^a^	-	-	-	2.97	0.001	-
	g/kg BW^0.75^	19	210.4 ^a^	-	218.4 ^a^	170.0 ^b^	208.0 ^a^	-	-	-	16.33	0.001	-
	g/kg DMI	21	1.247	-	1.311	1.155	1.255	-	-	-	0.08	0.146	-
Milk fat	g/kg	21	41.52 ^b^	-	48.22 ^a^	44.23 ^ab^	42.54 ^b^	-	-	-	3.48	<0.001	-
Milk protein	g/kg	17	32.97	-	39.84	31.84	32.55	-	-	-	3.08	0.084	-
Milk lactose	g/kg	12	46.92	-	50.11	48.08	46.64	-	-	-	2.19	0.339	-
Digestibility													
DMD	g/kg	40	700.4	730.5	717.02	705.7	697	-	683.4	-	29.41	0.491	0.014
OMD	g/kg	57	687.5	717	699.1	692.9	682.8	703.2	677.9	663.8	33.11	0.408	0.003
CPD	g/kg	44	644.7	-	638.1	645.5	647.3	673.8	628.2		38.59	0.658	0.321
NDFD	g/kg	47	563.2	-	568.3	550.6	560.8	569.7	551.3	547.6	47.46	0.972	0.321
ADFD	g/kg	27	494.9	-	-	484	493.8	-	496	-	48.29	0.956	0.269
Rumen fermentation parameters													
pH		148	6.28	6.09	6.26	6.11	6.31	6.14	6.8	-	0.26	0.341	0.440
NH_3_	mg/dL	146	23.6	27.19	19.14	21.83	23.26	23.66	17.42	19.78	6.64	0.386	0.224
Total VFA	mmol/L	74	104.9 ^a^	60.12 ^b^	116.5 ^a^	106.9 ^a^	111.1 ^a^	-	63.79 ^b^	-	57.83	<0.001	0.224
Acetate	%	71	63.34	64.14	29.99	66.45	63.75	-	60.36	66.32	10.27	0.106	0.798
Propionate	%	71	20.41	-	17.52	19.57	21.47	-	26.58	21.66	-	0.665	0.156
Butyrate	%	71	11.96	13.59	7.41	10.84	11.5	-	9.01	8.87	2.95	0.295	0.541
Valerate	%	29	1.34	-	0.9	-	1.44	-	1,17	-	0.42	0.782	0.126
A:P ratio		71	3.53	3.48	1.76	3.58	3.49	-	2.67	3.22	1.47	0.521	0.240
Protozoa	log_10_/mL	91	5.48 ^a^	5.17 ^b^	5.29 ^b^	5.63 ^a^	5.63 ^a^	5.33 ^b^	5.22 ^b^	5.02 ^b^	0.25	0.009	0.287
N Partitioning													
Urine N	g/kg BW^0.75^	35	619.9	622.8	521.9	637.1	615.0	597.5	-	570.84	51.20	0.614	0.245
Fecal N	g/kg BW^0.75^	35	572.6	557.2	590.1	567.9	574.8	584.8	-	554.91	46.47	0.997	0.001
N retention	g/kg BW^0.75^	23	463.2	-	537.3	368.5	486.2	523.5	-	459.46	49.82	0.188	-
Blood parameters													
Total protein	g/dL	20	6.66	-	7.4	6.63	6.2	7.85	-	-	0.95	0.279	0.689
Albumin	g/dL	13	2.92	-	3.24	3.1	2.91	-	-	-	0.22	0.308	-
Globulin	g/dL	10	3.64	-	4.20	3.51	3.32	-	-	-	0.35	0.228	-
PUN	mg/dL	72	18.07 ^b^	-	17.1335 ^b^	19.40 ^b^	17.90 ^b^	23.04 ^a^	-	-	1.20	0.001	0.345
Cholesterol	mg/dL	21	87.87	-	81.65	84.14	91.55	73.57	-	-	27.63	0.287	0.711
Creatinine	μmol/L	28	80.16 ^b^	-	87.67 ^b^	72.35 ^b^	80.14 ^b^	122.0 ^a^	-	-	12.29	<0.001	-
Glucose	mg/dL	20	53.65 ^a^	-	31.03 ^b^	53.56 ^a^	49.90 ^a^	31.62 ^b^	-	-	13.02	0.005	-
ALP	IU/L	10	170.8	-	-	175.9	161.9	-	-	-	32.69	0.388	-

DMI = Dry Matter intake; OMI = Organic matter intake; NDFI = neutral detergent fiber intake; BW^0.75^ = metabolic body weight; ADG = Average Daily Gain; DMD = dry matter digestibility; OMD = Organic matter digestibility; CPD = Crude Protein digestibility; NDFD = neutral detergent fiber digestibility; ADFD = acid detergent fiber digestibility; NH_3_ = ammonia; VFA = volatile fatty acids; A:P ratio = acetate propionate ratio; N = nitrogen; PUN = plasma urea nitrogen; ALP = alkaline phosphatase; SEM = standard error of the means; CON = Control diet, MS = *Medicago sativa*, QS = *Quilaja Saponaria*, CS = *Camelia sinensis*, YS = *Yucca schidigera*, AA = *Agave americana*, BP = *Biophytum petersianum*, SR = *Sapindus rarak*; ^a,b^ Different superscript within the row significant at *p* < 0.05.

## Data Availability

The dataset underpinning the results in the present study can be obtained from the corresponding author and will be considerable to share upon reasonable request.
